# Natural Deep Eutectic Extracts of Propolis, *Sideritis scardica*, and *Plantago major* Reveal Potential Antiageing Activity during Yeast Chronological Lifespan

**DOI:** 10.1155/2022/8368717

**Published:** 2022-08-30

**Authors:** Bela Vasileva, Dessislava Staneva, Tsvetinka Grozdanova, Hristo Petkov, Boryana Trusheva, Kalina Alipieva, Milena Popova, George Miloshev, Vassya Bankova, Milena Georgieva

**Affiliations:** ^1^Laboratory of Molecular Genetics, Institute of Molecular Biology “Roumen Tsanev”, Bulgarian Academy of Sciences, Sofia, Bulgaria; ^2^Institute of Organic Chemistry with Centre of Phytochemistry, Bulgarian Academy of Sciences, Sofia, Bulgaria

## Abstract

Nowadays, the environmentally friendly approach to everyday life routines including body supplementation with pharma-, nutraceuticals and dietary supplements gains popularity. This trend is implemented in pharmaceutical as well as cosmetic and antiageing industries by adopting a newly developed green chemistry approach. Following this trend, a new type of solvents has been created, called Natural Deep Eutectic Solvents (NADES), which are produced by plant primary metabolites. These solvents are becoming a much better alternative to the already established organic solvents like ethanol and ionic liquids by being nontoxic, biodegradable, and easy to make. An interesting fact about NADES is that they enhance the biological activities of the extracted biological compounds. Here, we present our results that investigate the potential antiageing effect of CiAPD14 as a NADES solvent and three plant extracts with it. The tested NADES extracts are from propolis and two well-known medicinal plants—*Sideritis scardica* and *Plantago major*. Together with the solvent, their antiageing properties have been tested during the chronological lifespan of four *Saccharomyces cerevisiae* yeast strains—a wild type and three chromatin mutants. The chromatin mutants have been previously proven to exhibit characteristics of premature ageing. Our results demonstrate the potential antiageing activity of these NADES extracts, which was exhibited through their ability to confer the premature ageing phenotypes in the mutant cells by ameliorating their cellular growth and cell cycle, as well as by influencing the activity of some stress-responsive genes. Moreover, we have classified their antiageing activity concerning the strength of the observed bioactivities.

## 1. Introduction

The World Health Organization (WHO) reports state that by 2050 the population of people over the age of 60 would double [1]. Though the real healthspan does not change a lot, yet older people are suffering from age-related diseases like cardiovascular, metabolic, and neurodegenerative, and many of them die because of them [2]. Ageing is defined by a decline in functional ability, dependent on time [3]. Many factors can contribute to the process like environmental and genetic influences and lifestyle. Many theories for ageing have been proposed, which are trying to describe the process [4]. For example, one of the most famous ones is the free radical theory, linking the accumulation of cellular damage with an increase of free radicals in the cells with age [5]. Lately, the epigenetics-focused “Information Theory of Ageing (ITA)” has been introduced [6–9], linking epigenetic changes with sequential switching on and off of certain genes that in turn lead to chromatin structure alterations, gene expression disbalance, and decreased DNA stability, all of which proven to speed up the ageing process. There have been numerous efforts to increase the maximum lifespan, and as the years have passed, with the modernization of healthcare and scientific advances, the human lifespan has tremendously improved. Therefore, the emphasis is on how to postpone or prevent ageing most healthily. And here, natural nutraceuticals are gaining attention [10].

The modern area in the organic chemistry extraction field is the new type of solvents called Natural Deep Eutectic Solvents (NADES), designated also as green solvents [11, 12]. What distinguishes them from the already known and massively used solvents is their production origin. NADES are made by mixing natural ingredients, mainly plant primary metabolites like organic acids, sugars, and amino acids. Interestingly, NADES exhibit a lower melting point, compared to one of the separate ingredients used to make them, and they are liquid at room temperature [13]. NADES are an important part of the so-called green chemistry branch because they are eco-friendly, easy to make and inexpensive, biodegradable, and nontoxic, compared to the currently used solvents like ionic liquids, ethanol, and methanol [13–15]. In addition to these beneficial properties, NADES have also been shown to increase the biological properties of the extracted compounds [16]. Recently, it has been shown that NADES solvents extract different natural compounds with potential antiviral and antimicrobial properties that could be used in medicinal practice [17, 18]. Moreover, recent papers reported on NADES extraction of polyphenols as a much safer and more efficient extraction approach [19, 20]. Polyphenols are well-known and profoundly studied antiageing factors with strong antioxidant and antimicrobial properties [21–24].

It is known that ageing is triggered and accompanied by dynamic changes in chromatin structure [25–27]. Moreover, chromatin plays a key role in the ageing process and the maintenance of its proper structure is crucial for the fate of the organism [28]. To provide access to DNA, chromatin undergoes remodelling through various types of mechanisms [29]. In addition, the state of chromatin can be altered by environmental stimuli, which subsequently affect the expression of genes associated with ageing and longevity [30, 31]. Much of the data we know nowadays about ageing comes from different model organisms like yeast, roundworms, fruit flies, and mice [32–34]. The reasons why these models are preferred are shorter lifespans, the ability for close follow-up, large-scale genetic screening, and functional genomics [35–37]. One of the most widely used models for ageing research is the yeast *Saccharomyces cerevisiae* [38, 39]*. S. cerevisiae* is considered the golden model for ageing due to its high similarity with other organisms, including humans [38, 40]. For example, cellular homeostasis, metabolism regulating signalling pathways, organelle functioning, cell growth and division, stress response, cell death, and ageing are highly conserved among eukaryotes and many are studied in the yeast cells as a pioneer model organism [39, 41]. Moreover, *S. cerevisiae* yeast is unique as it allows separate studying of the replicative (RLS) and chronological (CLS) lifespans [33]. The last is impossible in the other eukaryotes, especially humans. The RLS is defined by the number of the produced daughter cells during the period of the lifespan [33], referred to as the ageing of actively dividing cells. In contrast, the CLS is the ageing of nondividing cells that have entered the stationary phase [42]. These two models of ageing, RLS and CLS, are used to study how mitotically active and postmitotic cells age.

In this study, we are showing the perspective of using NADES extracted biocompounds in prospective antiageing therapies. We have tested the effect of two well-known Bulgarian medicinal plants—*Sideritis scardica* Griseb. (*S. scardica*) and *Plantago major* L. (*P. major*), as well as propolis (a compound produced by bees). Propolis is an established ingredient, particularly in the cosmetics industry for its antiageing properties [43, 44]. It has a high content of phenolic compounds and flavonoids and has been shown to influence the proliferation of fibroblasts [43]. *S. scardica* is a popularly used remedy in natural medicine due to its anti-inflammatory properties [45]. It is endemic to the Balkan peninsula mountain region [46]. There also have been reports on its antioxidant activity [23]. The third examined plant—*P. major*—is also rich in polyphenol content. It is also a well-known herbal medicine drug with established anti-inflammatory properties [47]. We have followed the CLS of four *S. cerevisiae* strains: the WT control and its isogenic chromatin mutants: *hho1delta* (without the gene for the linker histone), *arp4* (with a point mutation in the *ARP4* gene), and the double mutant *arp4 hho1delta* (which harbours the two mutations) [30, 48]. The four strains have been previously reported as a model for studying the role of chromatin organization in ageing [30, 31]. We have proved that the abrogated healthy interaction among the linker histone Hho1p and Arp4p leads to premature ageing phenotypes [30], which we now study in the presence of the tested NADES plant and propolis extracts. Our results demonstrate a potential antiageing activity of these NADES extracts proved by their ability to confer the premature ageing phenotypes in the mutant cells.

## 2. Materials and Methods

### 2.1. Preparation of NADES CiAPD14 Solvent

The citric acid (Chem-Lab NV, Zedelgem, Belgium) and 1,2-propanediol (Valerus, Sofia, Bulgaria) in a 1 : 4 molar ratio were mixed and stirred in a water bath (300 rpm) at 50°C until a homogeneous liquid was formed [19].

### 2.2. Extraction of Plant and Propolis Bioactive Compounds with NADES CiAPD14 Solvent

Row propolis was ground by a coffee mill after freezing. Plant materials (air-dried) were ground in a coffee mill. 50 mg of ground material (propolis/plant material) was extracted with 1.5 mL CiAPD14 in a 2 mL Eppendorf tube in an ultrasound bath (Elmasonic S 30 H), without heating for 1 h. The extracts were then centrifuged (13,000 rpm, 40 min) and filtered through cotton.

In the text, the NADES solvent CiAPD14 in its pure form will be referred to as CiAPD14, and the extracts made with it are as follows: CiAPD14 propolis, CiAPD14 *S. scardica*, and CiAPD14 *P. major*.

### 2.3. Quantitative Determination of Total Phenolics and Total Flavonoids

Total phenolics and total flavonoids were measured using previously reported methods (details in [19]). For blank: a solution of CiAPD14 instead of the test sample (CiAPD14 extract) was used in analogous procedures. For propolis, total phenolic content was estimated using a calibration curve of standard mixture pinocembrin–galangin 2 : 1 (*w*/*w*), and total flavonoid content was estimated using a calibration curve of galangin for the medicinal plants, calibration for total phenolics was performed using caffeic acid, and for total flavonoids with routine as the standard. Assays were carried out in triplicate.

### 2.4. *Saccharomyces cerevisiae* Strains

- Wild type (DY2864, WT): MATa *his4-912δ-ADE2 his4-912δ lys2-128δ can1 trp1 ura3 ACT3* [49]
- *hho1Δ* (*HHO1* KO derivative of DY2864, in the text appears as *hho1delta*): MATa *his4-912δ-ADE2 his4-912δ lys2-128δ can1 trp1 ura3 ACT3 ypl127C::K.L.URA3* [50]
- *arp4ts26* (DY4285, *arp4* mutant designated in the text as *arp4*): *MATa his4-912δ-ADE2 lys2-128δ can1 leu2 trp1 ura3 act3-ts26* [49]
- *arp4ts26hho1Δ* (double mutant derivative of DY4285, denoted as *arp4 hho1delta*): *MATa his4-912δ-ADE2 lys2-128δ can1 leu2 trp1 ura3 act3-ts26 ypl127C::K.L.URA3* [51]

### 2.5. Culture Media

- Yeast Extract–Peptone–Dextrose (YPD) liquid medium: 1% yeast extract, 2% dextrose, and 2% peptone (*w*/*v*)
- YPD-solid: 1% yeast extract, 2% dextrose, 2% peptone, and 1.5% agar (*w*/*v*)
- Synthetic complete medium (SC): 2% dextrose; 1.7% yeast nitrogen base and supplements (lysine, adenine, histidine, tryptophan, uracil, and leucine) according to the auxotrophic requirements of the strains

### 2.6. Yeast Cell Culture Growth Spectrophotometrical Measurements

The four yeast strains (1 × 10^7^ cells/mL) were cultivated in SC medium in a water bath shaker at 30°C for a CLS of nine consecutive days. Aliquots of yeast cell cultures were taken at four different time points—4^th^, 24^th^, and 72^nd^ hour and 9^th^ day of cultivation. For each, the optical density (OD_600_) was measured, and the obtained data were used for growth curve analysis.

### 2.7. Chronological Survival after Treatment with NADES Solvent CiAPD14 and NADES CiAPD14 Extracts with Propolis, *S. scardica*, and *P. major*

Control yeast cells and cells supplemented with CiAPD14 at a concentration of 0.089 (%*v*/*v*, *μ*L) and with propolis, *S. scardica*, and *P. major* CiAPD14 extracts at concentrations of 0.027, 0.046, and 0.045 (%*v*/*v*, *μ*L), respectively, were cultivated for nine days in SC medium. Aliquots were taken at three time points of the CLS (24^th^ and 72^nd^ hour and 9^th^ day), and 10^2^ cells were plated on YPD agar plates in triplicates and incubated for three days at 30°C. The number of plated cells was calculated by LUNA-II™ Automated Cell Counter (Logos Biosystems, USA) as well as following previously developed protocols for cell number assessment for these cultures based on their OD_600_. The percentage of viable cells in each group was determined by comparison of the number of colony-forming units (CFU) at the 72^nd^ hour and 9^th^ day time points with the number of CFUs at the 24^th^ hour of cultivation, which was perceived as 100%. The results are represented in graphs showing the cell survival percentage as MEAN ± SD.

### 2.8. Cell Cycle Analysis

Cell cycle analysis using fluorescence-activated cell sorting (FACS) was performed according to [50]. Briefly, aliquots were taken on the 4^th^, 24^th^, and 72^nd^ hour and 9^th^ day of cultivation and fixed in 96% ethanol at -20°C overnight. Following the next day, cells were centrifuged, resuspended in 50 mM sodium citrate, pH 7, and sonicated at 50% power for 20 seconds, after which they were incubated at 37°C for 30 minutes with 0.25 mg/mL RNAse A. Cells were stained with propidium iodide (50 *μ*g/mL) and acquired by a BD FACSCanto apparatus. Data analyses were performed using FlowJo V10 software.

### 2.9. Gene Expression Analysis

Total RNA was extracted from untreated cells as well as treated with NADES solvent CiAPD14 and NADES CiAPD14 extract-supplemented cells of the four strains at the four time points (4^th^, 24^th^, and 72^nd^ hour and 9^th^ day of cultivation) using the phenol-chloroform extraction protocol, according to [30]. 280 ng of total RNA, treated with DNase I, was then reverse transcribed into cDNA, using NG dART RT-PCR kit (EURx). The expression of three genes was examined in the cells, both in and without the presence of the tested bioactive NADES CiAPD14 extracts. The two genes of interest were *CDC28* and *RAD9*. *ACT1* was used as a reference gene. Primer sequences are shown in Table 1. Results were analyzed using Rotor-Gene 6000 software (Corbett Life Science) and calculated by the *^ΔΔ^*C_T_ method [52].

### 2.10. Statistical Analysis

All experiments were performed in triplicates. The statistical analysis for comparison of control (untreated) cells and treated with the extracts was performed in Excel, using the paired-samples *T*-test, two-tailed distribution. Results are represented as MEAN ± SD. Statistically significant results are denoted with ∗, where *p* < 0.05.

## 3. Results and Discussion

The potential antiageing effect of the NADES solvent CiAPD14 (citric acid : 1.2-propanediol in a ratio of 1 : 4) and the bioactive extracts CiAPD14 propolis, CiAPD14 *S. scardica*, and CiAPD14 *P. major* was studied during the chronological ageing of four *S. cerevisiae* strains: WT, *hho1delta*, *arp4*, and *arp4 hho1delta*, controls (nonsupplemented) and supplemented with the solvent and its three extracts. For each of the three mutant strains, we have previous data that evidenced their impaired chromatin organization and premature ageing phenotypes [30, 31, 48]. Briefly, the *arp4* mutant bears a point mutation in the gene coding for Arp4p—a subunit of INO80, NuA4, and SWR1 chromatin remodelling complexes, crucial for the proper chromatin organization and function [49]. The *hho1delta* mutant cells lack the gene *HHO1*, coding for the linker histone, which is responsible for chromatin higher-order structure organization [50]. The third mutant is *arp4 hho1delta*. It combines the two above-discussed mutations: the gene for the linker histone is knocked out and there is a point mutation in the *ARP4* gene [48]. We have previously studied these strains and proved that they work as a perfect model for studying ageing phenotypes [30, 31, 48, 54, 53].

The concentrations of the solvent CiAPD14 and the extracts CiAPD14 propolis, CiAPD14 *S. scardica*, and CiAPD14 *P. major* for yeast cell culture supplementation during the CLS were considered in our previous work and are listed in Table 2 [19].

### 3.1. Cell Growth Analysis of WT, hho1delta, arp4, and arp4 hho1delta Yeast Strains

**Figure 1:**
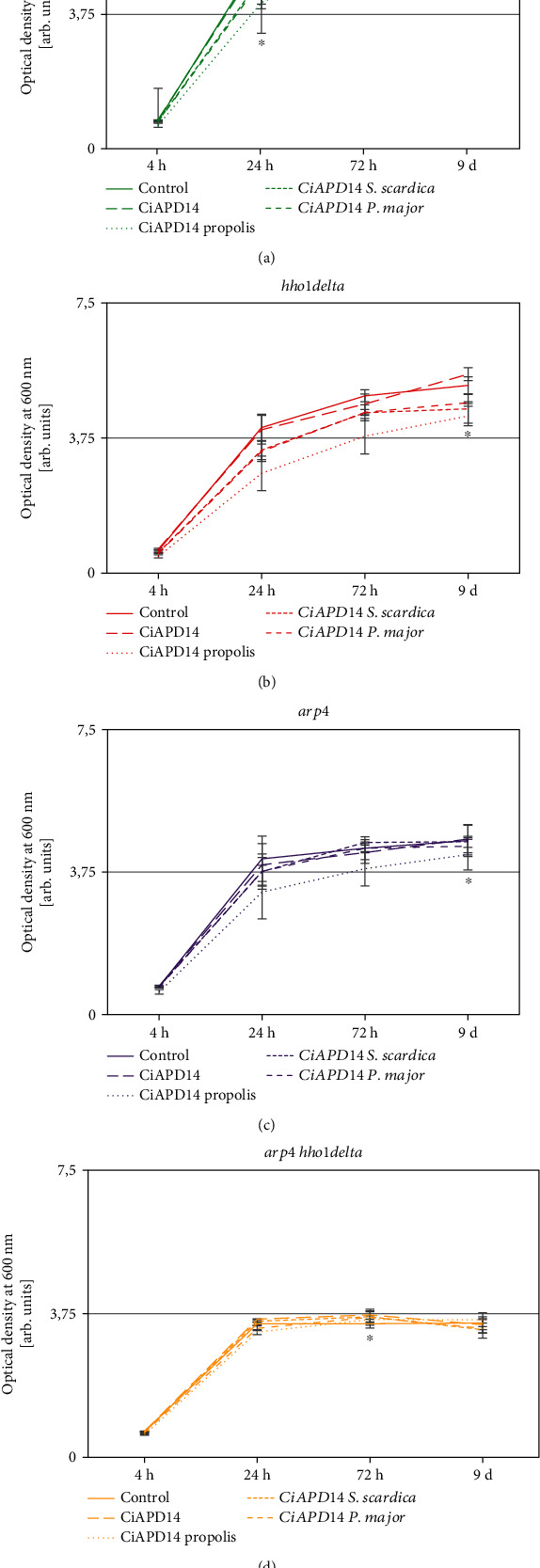
Cell culture growth of *WT*, *hho1delta*, *arp4*, and *arp4 hho1delta* without and with the presence of CiAPD14 NADES solvent and the three extracts. Cells were cultivated for the period of 9 days in SC media, supplemented with the studied NADES solvent and extracts. The control cells were not supplemented. At four time points: 4 h, 24 h, 72 h, and 9 d, aliquots were taken from the cell cultures and were analyzed spectrophotometrically. The optical density was measured in arb. units. Data are MEAN ± SD. Statistically significant differences between the growth of supplemented yeast cells compared to nonsupplemented are given with asterisk (∗); (a) WT, where ∗ means *p* < 0.05 for CiAPD14 *P. major*; (b) *hho1delta*, where ∗ means *p* < 0.05 for CiAPD14 propolis; (c) *arp4*, where ∗ means *p* < 0.05 for CiAPD14 propolis; (d) *arp4 hho1delta*, where ∗ means *p* < 0.05 for CiAPD14.

The studied yeast cells were cultivated in SC media for nine days. The control group of each yeast strain was not supplemented with the NADES solvent CiAPD14 or with any of the extracts. The supplemented yeast cell cultures were grown from day 1 of their CLS in SC media supplemented with CiAPD14, CiAPD14 propolis, CiAPD14 *S. scardica*, and CiAPD14 *P. major* at concentrations, which we have already designated as the half-maximal inhibitory concentrations (IC_50_). These IC_50_ data came from recent tests for cyto- and genotoxicity of the studied NADES solvent and extracts [19]. Table 2 summarizes these data as well as the data for the total phenolic concentration in the extracts. The cellular growth of the studied control and supplemented yeast cells was first explored by spectrophotometric measurements of the optical density of the cell cultures at a wavelength of *λ* = 600 nm (OD_600_). Cell culture aliquots of each of the four yeast strains were taken on the 4^th^, 24^th^, and 72^nd^ hour and 9^th^ day of cultivation in SC media for both control cells and treated with the solvent CiAPD14 and its three extracts: CiAPD14 propolis, CiAPD14 *S. scardica*, and CiAPD14 *P. major*. The results are presented as growth curves in Figure 1 (nontreated and cells supplemented with CiAPD14 or CiAPD14 propolis, CiAPD14 *S. scardica*, and CiAPD14 *P. major*). It is generally accepted that the chronological ageing of the yeast cells starts around the 24^th^ hour of cultivation [33]. Considering the chosen time points, our previous results from the investigation of the CLS of the three chromatin mutant strains have shown that up to the 9^th^ day of cultivation different premature ageing traits were detectable, which allowed us to test whether the NADES solvent CiAPD14 and its extracts CiAPD14 propolis, CiAPD14 *S. scardica*, and CiAPD14 *P. major* would exert antiageing properties [30, 31, 53].

First, to prove these yeast strains as a good model system for our studies, we compared the CLS cell culture growth of the control (untreated) yeast cells from the four strains. This analysis showed that WT yeast cells had the fastest cell growth during the fermentation period, i.e., between the 4^th^ hour time point and the 24^th^ hour (Figure 1(a)), whereas for the double mutant the growth was reduced by 20% (5.5-fold) (Figure 1(d)). During the stationary phase (from the 24^th^ hour to the 9^th^ day), we observed that for this period the WT cells increased furthermore their growth by 17% (Figure 1(a)), followed by the two single mutant cell cultures—*hho1delta* and *arp4*, respectively, with 21% and 10% (Figures 1(b) and 1(c)). The double mutant though did not show any increase in its growth for the observed period (Figure 1(d)). Compared to the other three yeast strains, the double mutant cell growth remained unchanged after the 24^th^-hour time point. These results indicated that at the 24^th^ hour the double mutant *arp4 hho1delta* was already in the stationary phase. A possible explanation was the premature ageing of these cells as these standard growth curves coincided with our previously published results [30, 31].

We further tested the solvent CiAPD14 and its extracts CiAPD14 propolis, CiAPD14 *S. scardica*, and CiAPD14 *P. major* on the cell culture growth of these cells (Figure 1, dotted lines). Interestingly, in all supplementations of the cells, we did not observe a statistically significant increase in the optical density when compared to the nontreated cells. On the contrary, the cell growth was slightly slower in comparison to the untreated cells. The only statistically significant difference in the slower growth of supplemented cells was detected in the growth of the WT, supplemented CiAPD14 *P. major* at the 24^th^ hour (Figure 1(a), ^∗^*p* < 0.05), for *hho1delta* and *arp4* cells supplemented with CiAPD14 propolis on the 9^th^ day (Figures 1(b) and 1(c), ^∗^*p* < 0.05), while for the double mutant (Figure 1(d), ^∗^*p* < 0.05) statistically significant appeared the detected slower growth of these cells with the solvent only.

In summary, we did not observe any notable changes in the cell culture growth of the studied yeast cells, supplemented with CiAPD14 and the three extracts. We consider this a positive result, especially in the light of the CLS of these cells as cell culture growth measured spectrophotometrically does not directly reflect the ageing of the yeast cells. Moreover, it is a sign of biocompatibility of the NADES solvent and its extracts and is a good prerequisite for the following experiments that target the in-depth study of the CLS of these cells in the presence of the studied NADES compounds.

### 3.2. Chronological Lifespan of Four Yeast Strains in the Presence of NADES Solvent and Extracts

Standard protocols for the study of chronological ageing in *S. cerevisiae* include observing the survival of cells at different time points from their growth in SC media. The presumption is that the preserved viability of cells, in particular, the ability to divide after being plated on a solid rich culture medium, is evidence for the progression of their chronological age [32, 48]. To monitor and analyze the cell survival of the four studied yeast strains, the cells were cultured in SC media for 9 days. The nonsupplemented cells of each strain served as controls. Three time intervals were analyzed: 24^th^ and 72^nd^ hour and 9^th^ day. The analysis of the chronological lifespan was determined by counting the colony-forming units (CFUs) and calculating the cell survival of each strain at the given time point as a percentage of viable cells (CFUs) at the 24^th^ hour of cultivation, taken as 100%. The 24^th^-hour time point is the period when the yeast cells enter the stage of the so-called diauxic shift (the time when fermentation stops as a result of depletion of glucose in the medium) and respiration begins [54, 55]. This is the moment when the yeast chronological ageing commences [48, 53].

**Figure 2:**
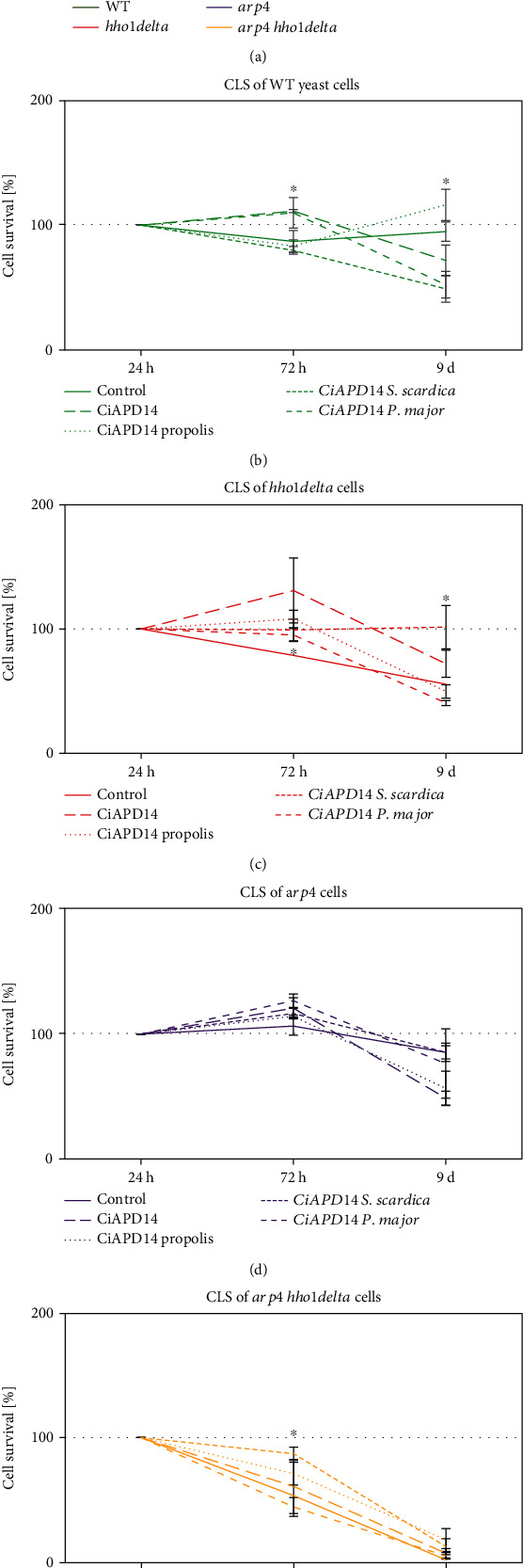
CLS of WT, *hho1delta*, *arp4*, and *arp4 hho1delta* in the presence of CiAPD14 and the three extracts. Cells were cultivated for the period of 9 days in SC media supplemented with the studied NADES solvent and extracts. At three time points: 24 h, 72 h, and 9 d 100, cells were taken from the cell cultures and were seeded on rich solid YPD media. Cells were allowed to recover under optimal conditions (30°C). The cell survival at the 24^th^-time point was assumed as 100%, and the survival of cells at all other time points were calculated as a percentage of it. (a) CLS of the four studied strains without any treatment; (b) CLS of the WT cells, control and supplemented with CiAPD14 and extracts; (c) CLS of *hho1delta* mutant cells, control and supplemented with CiAPD14 and extracts; (d) CLS of *arp4* mutants; (e) CLS of the double *arp4 hho1delta* control cells and in the presence of CiAPD14 and extracts. Data are MEAN ± SD. Statistically significant differences are marked with ∗ where *p* < 0.05.

First, we analyzed how the four strains survived without being supplemented (Figure 2(a)). The results showed that the wild type maintained a stable CLS until the last monitored time point with 87% and 94% viable cells on the 72^nd^ hour and 9^th^ day, respectively. The *arp4* mutant had the highest percentage of viable cells throughout its CLS, which on the last point dropped to only 15% (85% viable cells in comparison to the 24^th^ h time point). The survival rate of the *hho1delta* gradually decreased in the course of CLS showing a reduction of 21% and 44% at 72^nd^ h and 9^th^ d, respectively (^∗^*p* < 0.05). As for *arp4 hho1delta*, its cells were short-lived and had a strong decrease with an almost 98% drop in their survival rate from the 24^th^ h time point till the 9^th^ day (^∗^*p* < 0.05). The abrogated survival rate detected for the double mutant *arp4 hho1delta* cells was proven by our previous results [30].

Figure 2(b) shows the CLS cell survival rates obtained for the control WT cells after treatment with CiAPD14 and with the three extracts. The graph clearly shows that after treatment with CiAPD14 or with CiAPD14 *P. major*, the viability at the 72^nd^ hour increased by 10% compared to the initial time point and by 20% compared to the nonsupplemented cells (^∗^*p* < 0.05), with a subsequent decrease by 39% and 58%, respectively, on the 9^th^ day. In the cells treated with CiAPD14 propolis, there was a significant increase in the percentage of colonies obtained on the 9^th^ day, in comparison to the percentage of colonies in the untreated cells (^∗^*p* < 0.05). An increased cell survival during chronological ageing was also observed in the WT yeast cells treated with CiAPD14 *P. major*. There was an increase of 23% in the percentage of colonies formed at the 72^nd^ hour for CiAPD14 solvent (nonsignificant) and CiAPD14 *P. major* (^∗^*p* < 0.05) supplemented WT cells when compared to the percentage of viable cells obtained in the nonsupplemented ones.

The results obtained for the *hho1delta* mutant (Figure 2(c)) differed from the data reported for the wild type, as on the last time point certain antiageing effects were observed for the CiAPD14 solvent and its extract with *S. scardica*, which was the most pronounced. In the *hho1delta* cells, treated with the CiAPD14 S*. scardica* extract, the cell survival at all-time points was around 100% (^∗^*p* < 0.05 for the 9^th^ day). The CiAPD14 propolis and CiAPD14 *P. major* supplementation had a beneficial effect, i.e., increased survival percentage only at the 72^nd^ hour representing the early stage of chronological ageing (^∗^*p* < 0.05).

The data reported for the *arp4* mutant (Figure 2(d)) showed no statistically significant differences in the cell survival of these cells among supplementations with different extracts, whereas the results obtained for the *arp4 hho1delta* strain (Figure 2(e)) showed that the highest percentage of cell survival appeared at the 72^nd^ hour after supplementation of these cells with CiAPD14 S*. scardica* (34% increase in comparison to the same strain but nonsupplemented, compare Figures 2(a) and 2(e), 72^nd^ h, where ^∗^*p* < 0.05). Only 2% of cells of the double mutant survived on the 9^th^ day if they are untreated; however, the supplementation with CiAPD14 propolis and CiAPD14 S*. scardica* increased insignificantly the viability of these cells on the 9^th^ day to 18% and 12.8%, correspondingly (Figure 2(e)). We understand that these differences in the CLS of the double mutant are not so explicit on day 9^th^ with CiAPD14 propolis and CiAPD14 *S. scardica*, but we discuss them here as they highlight their potential antiageing properties. Consider that these cells did not preserve their ability to divide at day 9^th^ unsupplemented, then even 16% and 10% induction of it means potential for stimulating the division of these cells and deserves our attention.

Therefore, we conclude that CiAPD14 pure (for the *hho1delta* mutant, Figure 2(c), ^∗^*p* < 0.05), CiAPD14 propolis, and CiAPD14 *S. scardica* (especially for the double mutant cells at the 72^nd^ h and 9-day time points, Figure 2(e), ^∗^*p* < 0.05) showed the most pronounced antiageing effect on the yeast cells. These results support the idea that the tested CiAPD14 extracts had antiageing activity on the prematurely aged mutant cells as well as in the single mutants that lack the linker histone and merit further investigations [50]. The results obtained for the supplemented with the three extracts cells roughly followed the measured total phenolic content in these extracts, where the CiAPD14 propolis extract has the highest phenolic content, followed by the CiAPD14 *S. scardica* and CiAPD14 *P. major* extracts (Table 2).

### 3.3. Analysis of Yeast Cell Cycle after Supplementation with NADES Extracts

**Figure 3:**
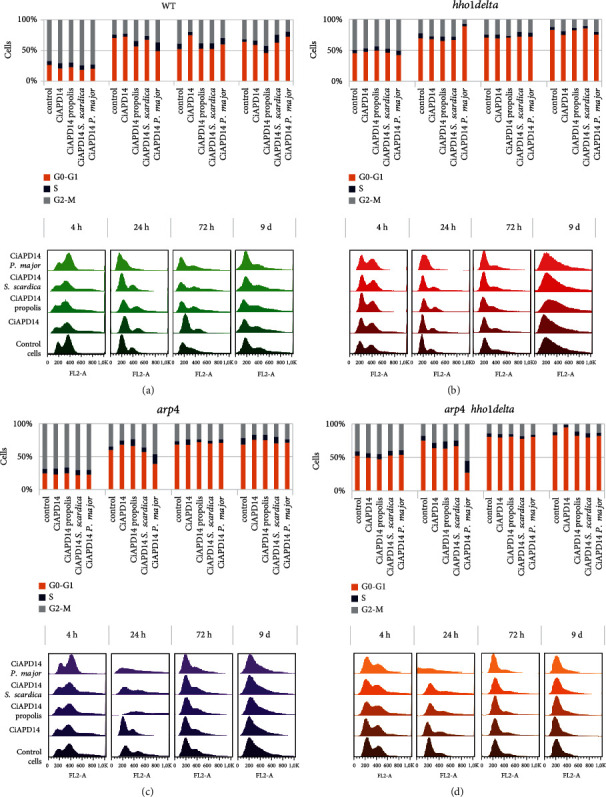
Cell cycle analysis of the four studied strains WT, *hho1delta*, *arp4*, and *arp4 hho1delta* in the presence of CiAPD14 and the three extracts during their CLS. Cells were cultivated during the CLS for nine days. At four time points: 4 h, 24 h, 72 h, and 9 d, aliquot cells were taken from the cell cultures and were analyzed by FACS after propidium iodide staining. (a) WT strain FACS data quantitation (bar chart) and representative histograms (below) for each time point; (b) *hho1delta* strain FACS data quantitation (up) and representative histograms for each time point; (c) *arp4* FACS data quantitation (up, bar chart) and representative histograms for each time point; (d) *arp4 hho1delta* FACS data quantitation (up) and representative histograms for each time point. Data are represented as % of cells in each phase of the cell cycle.

An important measure of yeast chronological ageing is cell progression through the cell cycle phases. Typically, after entering the stationary phase, which generally happens at the 24^th^ hour of cultivation, the majority of cells become quiescent [56]. Having this in mind, we tested whether CiAPD14 or the extracts would affect the cell cycle of the studied strains. We cultivated the four *S. cerevisiae* strains WT, *hho1delta*, *arp4*, and *arp4 hho1delta* in SC media for 9 days. Cells were supplemented with CiAPD14 and the NADES extracts of CiAPD14 propolis, CiAPD14 *P. major*, and CiAPD14 S*. scardica* at the studied concentrations (see Materials and Methods) from the 1^st^ day of their cultivation in SC media. Aliquots were taken on the 4^th^, 24^th^, and 72^nd^ hour and 9^th^ day of cultivation. Cells were washed and stained with propidium iodide and subsequently analyzed by FACS. FACS results are represented as both percentages of cells in the cell cycle phases and descriptive histograms (Figure 3).

We first analyzed the way the untreated cells of the four strains chronologically aged by assessing the percentage of cells in the cell cycle phases (Figure 3, see the control in bar charts of each panel). We confirmed a similar trend found in our previous results. This trend resulted in the distribution of logarithmically growing yeast cells from the WT and *arp4* mutant (4^th^ h) predominantly found in the G2/M phase of the cell cycle (Figures 3(a) and 3(c), histograms, control cells), which is considered the normal way the yeast cell cycle goes at this time point [30, 31, 57, 58]. In our case, the two chromatin mutants that lack the *HHO1* gene encoding the linker histone demonstrated a visible shift of the cells from G2/M to G0/G1 cell cycle phase (4^th^ h) (Figures 3(b) and 3(d), representative histograms, controls). Following the rest of the time points, the control cells showed that the chronologically aged nonsupplemented cells from the four strains were mainly in the G0/G1 phase of the cell cycle, which was especially prominent on the last day of cultivation, the 9^th^ day [26, 58–60]. Moreover, according to previous reports [59, 60], after the diauxic shift, two distinguishable cell populations are presented in the G1 phase of stationary phase yeast *S. cerevisiae* cultures, quiescent (Q) population, in which daughter cells are predominant and are the longest-lived, and non-Q population, in which mother cells are predominant and are the shorter-lived. On day 9, the largest percentage of cells was in G1, which was most distinctive for the *hho1delta* and *arp4* h*ho1delta* strains and less pronounced for the WT. We could speculate that although stationary cultures of these mutants have а high percentage of G1 phase cells, they are rather non-Q, non-long-lived cells and therefore poor survivors. This in turn raises the question of the relationship between the ability of chromatin mutants to form a proper quiescent cell population and the chronological lifespan, thus highlighting the need for further research to elucidate it.

The yeast cells supplemented with CiAPD14 or CiAPD14 propolis, CiAPD14 *P. major*, and CiAPD14 *S. scardica* were also analyzed by FACS, and the results are demonstrated in Figure 3 (see supplemented cells).

For the WT cells (Figure 3(a)) there were different tendencies of transition of supplemented with the NADES extracts cells from G0/G1 into the G2/M and *vice versa* in the cell cycle phase from the 4^th^ to 72^nd^ hour of CLS, compared to the untreated control, which corresponded with the cell survival experiments (Figure 2(b)). CiAPD14 propolis-supplemented WT cells by the 9^th^ day of cultivation experienced the highest survival (Figure 2(b)) and the highest percentage of cells transiting through the cell cycle phases (Figure 3(a)). There was a tendency for predominant accumulation of cells in the G0/G1 phase of the cell cycle for the logarithmically growing mutants *hho1delta* and *arp4 hho1delta* that lack the linker histone gene *HHO1*, which did not change after treatment with the solvent, nor with any of the three plant extracts (Figures 3(b) and 3(d)). G0/G1 accumulation of cells is important for the longevity of cells though could be accepted as a positive and negative trait. On day 9, for all cultures studied, the largest percentages of cells were in phase G1. Moreover, we detected a correlation of the effect of CiAPD14 propolis extract which increased both the cellular viability and the percentage of G2/M cell fraction. We find these results intriguing and deserve our attention. In general, there was no bold cytostatic, nor proproliferative effect of the solvent and its extracts on any of these two mutant strains. Slight exceptions showing an increased % of cells in G0/G1 after treatment with CiAPD14 *P. major* were detected for the *hho1delta* cells at the 24^th^ h time point (Figure 3(b)). The results with *hho1delta* and the double mutant *arp4 hho1delta* confirmed that the lack of the linker histone highly influenced the CLS of the mutant cells and its absence was an obstacle for cells to cope with the ageing process, most probably due to their strongly abrogated chromatin structure [30, 48, 50, 53]. The above-discussed tendencies in cell cycle progression of the studied cells, non- and supplemented with the studied CiAPD14 solvent and extracts, are depicted with representative histograms for ease of comparison (Figure 3, in-built histograms, green—WT, red—*hho1delta*, purple—*arp4*, and orange—*arp4hho1delta*). This yet again verified how important were the linker histone and the proper chromatin organization for the ageing process.

### 3.4. RT-qPCR Analysis of the Expression of DNA-Damage Response Genes during CLS of the Yeast Cells with and without CiAPD14 and Extract Supplementation

**Figure 4:**
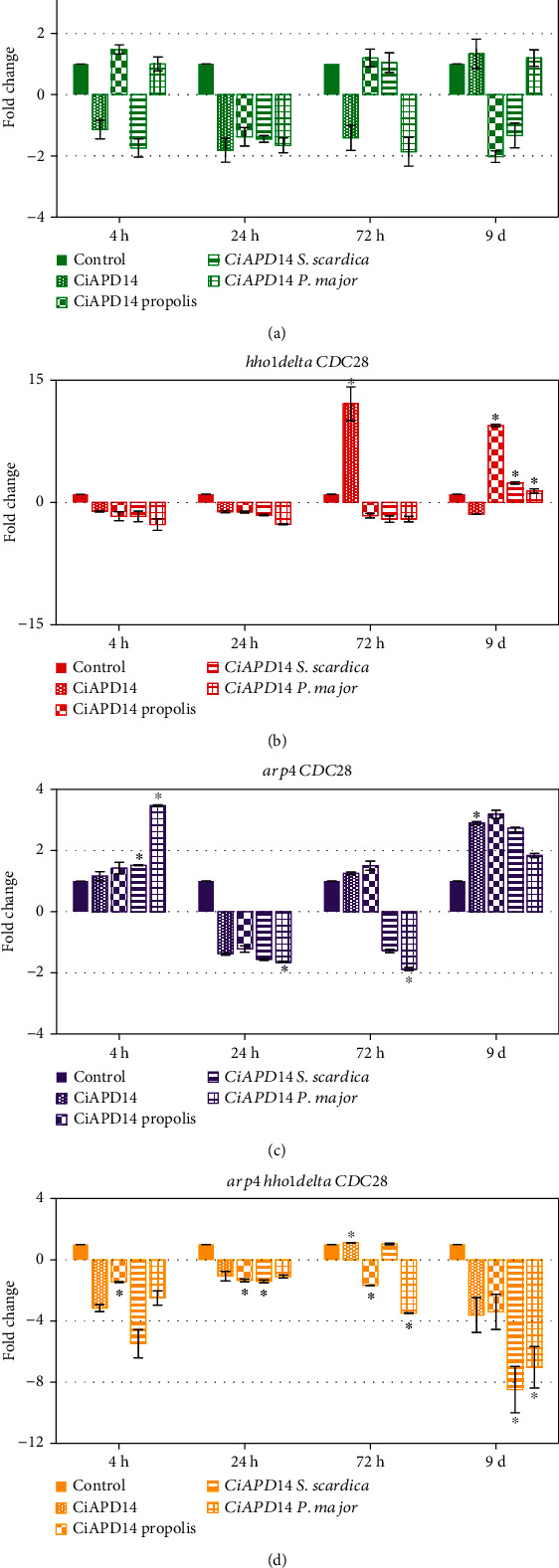
Gene expression analysis of the *CDC28* gene of treated with CiAPD14, CiAPD14 propolis, CiAPD14 *S. scardica*, and CiAPD14 *P. major* extract yeast cells. Total RNA was isolated from aliquots of the yeast cultures and converted to cDNA. cDNA was used as a template in RT-qPCR experiments to analyze the expression of *CDC28*. *ACT1* was expression the reference gene. (a) WT, (b) *hho1delta*, (c) *arp4*, and (d) *arp4 hho1delta*. The results were calculated by the *^ΔΔ^*CT method using specialized Rotor-Gene 6000 software. Data are MEAN ± SD. Statistically significant differences are marked with ∗ where *p* < 0.05.

**Figure 5:**
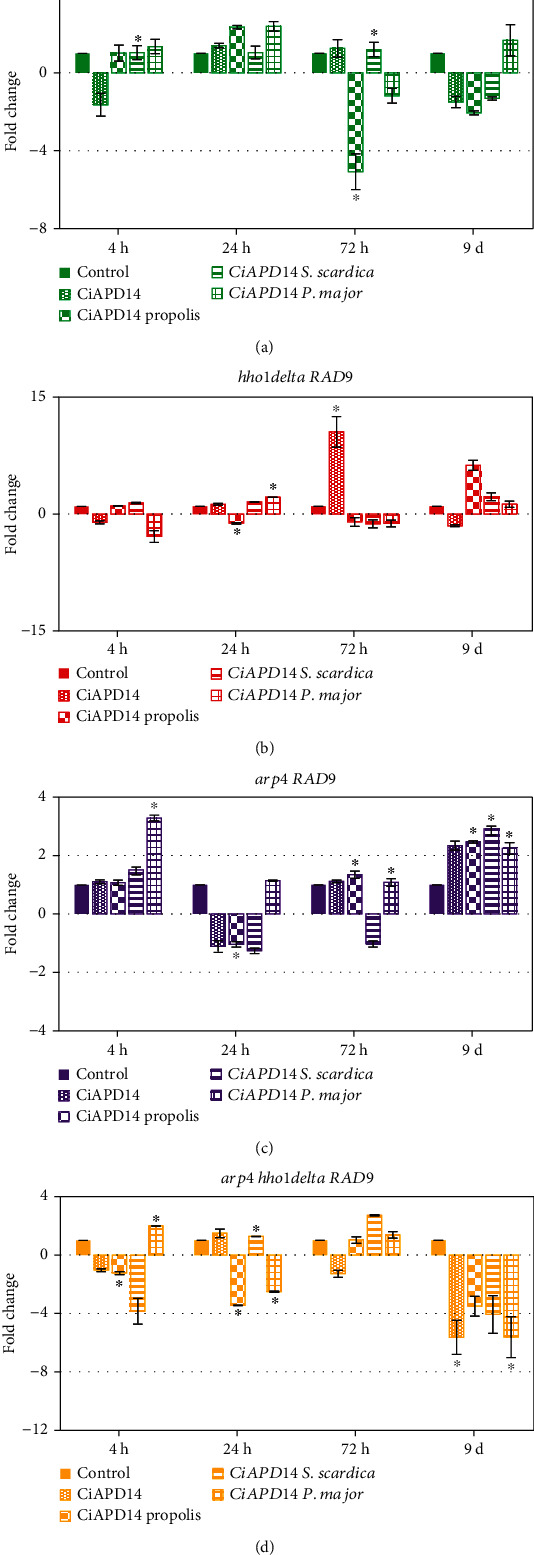
Gene expression analysis of the RAD9 gene of supplemented with CiAPD14, CiAPD14 propolis, CiAPD14 *S. scardica*, and CiAPD14 *P. major* extract yeast cells during their CLS. Total RNA was isolated from aliquots of the yeast cultures and converted to cDNA. cDNA was used as a template in RT-qPCR experiments to analyze the expression of *RAD9*. *ACT1* was the reference gene. (a) WT control cells, (b) hho1delta mutant yeast cells, (c) arp4 mutants, and (d) arp4 hho1delta double mutant cells. The results were calculated by the *^ΔΔ^*CT method using the specialized Rotor-Gene 6000 software. Data are MEAN ± SD. Statistically significant differences are marked with ∗ where *p* < 0.05.

During the experimental monitoring of the growth potential, cell survival, and cell cycle of yeast *S. cerevisiae* cells of the four strains, aliquots were also taken to track the gene expression of two key genes, *CDC28* and *RAD9*. Untreated yeast cells of each strain were used as a control group. To examine the gene expression of the selected genes, total RNA was isolated from aliquots of the yeast cultures and converted to cDNA. This cDNA was used as a template in experiments to analyze the expression of the two genes of interest with *ACT1* as a reference gene. The results were calculated by the *^ΔΔ^*CT method using specialized Rotor-Gene 6000 software. Primer pairs used in the RT-qPCR are shown in Table 1. We examined the expression of *CDC28* and *RAD9* genes in the course of the chronological ageing of the studied four yeast strains with and without supplementation with the CiAPD14 and with the three biological NADES extracts (Figures 4 and 5). The main role of the *CDC28* gene product is to regulate the mitotic and meiotic cell cycle. Cdc28p is also involved in the regulation of cellular metabolism, the maintenance of chromosome dynamics, cell growth, and morphogenesis. The gene is associated with a cell cycle block in the G2/M checkpoint [61]. Studies show that this gene is active during the G2/M cell cycle phase, compared to its relative inactivity in the G0/G1 phase, due to low cyclin expression and abundance in CDK inhibitors Sic1 and Far1 [61].

In our experiments, an insignificant decrease of *CDC28* gene expression (less than 2-fold) was observed in the four yeast strains when they entered the stationary phase at the 24^th^ hour of cultivation (see Figure 1) and had their cells predominantly in the G0/G1 cell cycle phase in all types of NADES supplementations (see Figure 3). These results were in agreement with the role of Cdc28p in the cell cycle regulation as discussed above. The studied WT yeast cells supplemented with CiAPD14 and extracts did not show any statistically significant changes in *CDC28* expression during all time points (Figure 4(a)). This was an indication that in the normally ageing cells the NADES extracts did not influence the expression of this gene as cells were able to moderate their ageing process. In the course of the chronological ageing, the *hho1delta* mutant showed the highest increment in *CDC28* expression of 12-fold, after treatment with CiAPD14 at the 72^nd^ hour, as well as an increase of 9-fold and 2.4-fold in *CDC28* expression after treatment with CiAPD14 propolis on day 9^th^ (^∗^*p* < 0.05). On the 9^th^ day, these cells supplemented with CiAPD14 *S. scardica* and CiAPD14 *P. major* also demonstrated a statistically significant increase (2-fold change, ^∗^*p* < 0.05) (Figure 4(b)). It is worth mentioning that the Cdc28 kinase is an important part of the homologous recombination process [62–64], for which the linker histone is an inhibitor [65]; therefore, the lack of Hho1p in the *hho1delta* mutant cells increased *CDC28* expression, especially on the last day of CLS and specifically after supplementation with CiAPD14 propolis, CiAPD14 *S. scardica*, and CiAPD14 *P. major*. Interestingly, the comparison of the results for the expression of this gene after supplementation with CiAPD14 propolis in WT and *hho1delta* cells at the 9^th^ day showed unchanged expression of *CDC28* for WT and increased by 9.4-fold for *hho1delta* mutant, which coincided with the high phenolic content of this extract (Table 2, propolis—3.3048 *μ*g/mL), but did not correlate with the CLS survival of these *hho1delta* mutant cells on day 9^th^ after CiAPD14 propolis supplementation (Figure 2(b)). We observed that indeed the CiAPD14 propolis extract led to the unchanged survival rate of the WT cells at the last time point for the *hho1delta* (compare Figures 2(b) and 2(c)). The last was an indication that *hho1delta* cells experienced stress on the 9^th^ day, which led to increased expression of *CDC28* and a slight recovery in the G0/G1 blockage of these cells after CiAPD14 propolis supplementation (Figure 3(b), see representative histograms for this strain). We speculate that the highest phenolic content in the CiAPD14 propolis probably led to the induction of oxidative stress, to which these mutant cells were intolerant [30]. This hypothesis is strengthened to an extent by the results with the supplementation of *hho1delta* cells with the CiAPD14 solvent only on the 72^nd^ h time point, where we detected a correlation between the *CDC28* elevated expression and the CLS survival rates of these cells at that particular time point probably due to lack of phenols in the solvent. We find these results interesting and a good groundwork for our future experiments for studying the underlying mechanisms of the potential antiageing properties of the tested NADES solvent and extracts.

*Arp4* cells experienced an increase in the relative amount of *CDC28* mRNA at the 4^th^ h and on the 9^th^ d after supplementation with CiAPD14 *P. major* and CiAPD14 *S. scardica* with a 3.5-fold and 2-fold change, respectively (^∗^*p* < 0.05) (Figure 4(c)). On the 9^th^ day of cultivation, there was an increment in the *CDC28* expression levels after treatment with CiAPD14 propolis, CiAPD14 *S. scardica*, and CiAPD14 *P. major* extracts with a 3.2-, 2.7-, and 1.8-fold change increase (^∗^*p* < 0.05) (Figure 4(c)). These results though statistically significant did not correlate with the CLS survival results of these cells (Figure 2(d)) as we did not detect any statistically significant differences among the CLS survival in the supplemented with the NADES solvent and its extracts cells and the nonsupplemented ones. We, therefore, find these results as a good basis for future experiments that aim at elucidation of the underlying mechanisms of the potential antiageing effect of the studied NADES solvent and its extracts. An interesting observation was that the double mutant had a general reduction in the expression of the *CDC28* gene in the cells supplemented either with the solvent or with an extract during all measured time points (Figure 4(d)), with the most pronounced decrease of 3.4- and 8.5-fold at the 9^th^ day (^∗^*p* < 0.05). The results of *CDC28* expression in the double mutant were in а good agreement with the results from the CLS and cell cycle experiments. It can be seen that the lower expression of *CDC28* in this mutant coincided with shorter CLS (Figures 1 and 2) and the detection of the fewest cells in the S and G2/M phase of the cell cycle at later points of CLS (Figure 4(d); 9^th^ day) compared to all other strains.

Next, we examined the expression of *RAD9* during the ageing of the studied yeast cells (Figure 5). *RAD9* is responsible for early DNA damage response and cell cycle checkpoint regulation [66, 67]. For the WT (Figure 5(a)), we did not observe any significant changes in the expression of the *RAD9* gene on the 4^th^ h for the control WT cells except after supplementation with CiAPD14 *S. scardica*, which led to a statistically significant slight increase in its expression (^∗^*p* < 0.05). On the 24^th^ hour, we did not detect any significant differences, while at the 72^nd^ hour, we noticed a 5-fold decrease (^∗^*p* < 0.05) of *RAD9* expression in the WT cells supplemented with CiAPD14 propolis and a twofold increase (^∗^*p* < 0.05) after CiAPD14 *S. scardica* supplementation. On the last 9^th^ day, none of the detected differences was statistically significant. *RAD9* expression in the *hho1delta* mutant (Figure 5(b)) on the 24^th^ hour had an increase in the cells supplemented with CiAPD14 *P. major* around 2-fold (^∗^*p* < 0.05). *RAD9* expression was increased, particularly after treatment with CiAPD14 solvent only for the 72^nd^ hour with 10-fold (^∗^*p* < 0.05). We detected a 6.3- and 2.2-fold increase after CiAPD14 propolis and CiAPD14 *S. scardica* treatment on the 9^th^ day, though the last two differences were statistically insignificant. Thus, as for *CDC28*, *RAD9* transcription was upregulated upon supplementation with CiAPD14 on the 3^rd^ day and upon supplementation with propolis and *S. scardica* extracts on day 9 of the CLS. Quite similar was the trend of *CDC28* and *RAD9* gene expression in the *arp4* cells supplemented with CiAPD14 or the CiAPD14 biological extracts in all examined time points. We did not detect any correlation between the CLS survival of these cells and the detected changes in the expression of *RAD9*. We assume that in some of our mutants the detected changes in the expression of the studied genes could require more time to be visualized as a change in the ageing process as the mutants are different and express different features of ageing. The double mutant had a general decrease in *RAD9* expression (Figure 5(d)) for cells supplemented with CiAPD14 propolis at the 4^th^ h time point (^∗^*p* < 0.05) and an increase of 3.5-fold for cells supplemented with CiAPD14 *P. major* (^∗^*p* < 0.05). On the 24^th^ hour, this tendency for CiAPD14 propolis-supplemented cells was preserved. A similar decrease in *RAD9* was observed for the cells supplemented with CiAPD14 *P. major* too (^∗^*p* < 0.05). Statistically significant differences were not detected in the expression changes of *RAD9* at the 72^nd^ hour in these cells. The most prominent decline was detected on day 9^th^, where all the treatments resulted in significantly lower *RAD9* expression levels. The statistically significant differences were detected for the supplementation of these double mutant cells with CiAPD14 solvent and CiAPD14 *P. major* (^∗^*p* < 0.05).

**Figure 6:**
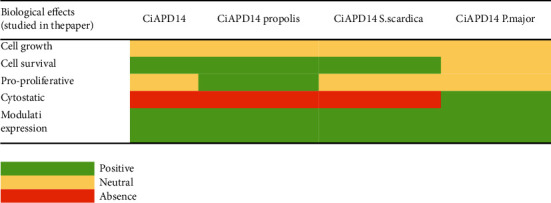
Combined general antiageing effects of CiAPD14 and its extracts for all the studied *S. cerevisiae* strains during their CLS.

We have summarized the obtained results of the potential antiageing properties of the tested NADES solvent and plant extracts in Figure 6. The effects are combined and evaluated for all yeast strains. The biological activities of the NADES solvent and extracts are combining data from the cell growth and survival assays, FACS analyses, and gene expression studies for all studied strains. This is done for the ease of classification of their antiageing properties.

What we observed was that the solvent itself (CiAPD14) exhibited slight antiageing properties (follow the green coloured boxes for CiAPD14 in Figure 6). This result was further acceptable given the fact that the solvent alone should not change the way cells age, though the combination of citric acid and 1,2-propanediol could induce certain biological effects as recent studies showed [68]. These data discussed that the citric acid exerted certain biological effects on the studied HaCaT cells by inhibiting their proliferation via the induction of cell cycle arrest and apoptosis. Treatment of cells with citric acid or malic acid led to apoptotic features, DNA damage, and an increase of sub-G1 cells, which was connected with cell regeneration.

The comparison among the overall antiageing activities of the studied CiAPD14 extracts showed that CiAPD14 propolis was the most potent one (follow the green boxes in Figure 6 for CiAPD14 propolis), followed by CiAPD14 *S. scardica* and CiAPD14 *P. major.* This classification based on the combined antiageing properties of the tested NADES extracts was in full compliance with the total phenolic content, presented in Table 2, and further confirmed the antiageing potential of these extracts. We believe that these results are another important indicator of the increasing need for a detailed evaluation of the mechanism of action of natural compounds and metabolites in biological processes.

## 4. Conclusions

NADES origin is completely natural as NADES solvents are made typically of plant primary metabolites, and one of their most important characteristics, apart from being nontoxic, is the fact that they have been proven to increase the beneficial properties of the extracted biological compounds, which gives them a new ground for future development of different therapies [18, 19]. In this study, we tested the potential antiageing effects of a novel NADES solvent—CiAPD14, as well as three biological extracts with it from propolis, *S. scardica*, and *P. major.* The advantages of our work come from the fact that this is the first report on the potential antiageing properties of a NADES solvent (CiAPD14) and its extracts with propolis, *S. scardica*, and *P. major*. We tested their antiageing properties on yeast *S. cerevisiae* cells, commonly accepted as a golden model organism for studying ageing. The chronological ageing of three chromatin mutants was studied with and without supplementation with the NADES extracts, which further enriched our studies as the chromatin mutants have disorganized chromatin structures resulting in premature ageing phenotypes [30, 31, 50, 53]. The results confirmed the potential of NADES for extraction of biologically active secondary metabolites from propolis and the studied medicinal plants and further suggested that NADES CiAPD14 could improve the effects of bioactive constituents of the extracts. This finding is important, because the recovery of the extracted compounds from the NADES, which are nonvolatile, is challenging. Further studies are needed to elucidate the role of the studied NADES on the bioactivity of dissolved substances and the possibility to use such extracts in the food industry and pharmacy. Our results showed that the solvent CiAPD14 did exert a slight antiageing effect, which was something that would not be typically expected, considering the solvent was in its pure form. When the three NADES extracts were tested, however, we noted articulate antiageing effects (Figure 6). The antiageing properties are reflected in the slowed course of their CLS, especially for the double mutant, in the transition of their cells in the cell cycle phases and the change in expression of genes involved in DNA repair pathways. We further detected changes in the cell proliferation of these cells supplemented with the NADES solvent and extracts. On the last time point tested for all cultures, the largest number of cells was in phase G1, which was most distinctive for the *hho1delta* and *arp4 hho1delta* strains and less pronounced for the WT. We hypothesized that although stationary cultures of these mutants have а high percentage of G1 phase cells, they are rather poor survivors. This in turn raises the question of the relationship between the ability of chromatin mutants to form a proper quiescent population and the chronological lifespan and the need for further research to elucidate it. Moreover, we detected a correlation of the effect of CiAPD14 propolis extract which increased both, the cellular viability and the percentage of G2/M cell fraction. Our unpublished data show that the yeast mutants that lack the gene for the linker histone have aberrant and decreased percentage of quiescent cells during the CLS, which results in their premature ageing phenotypes and this is further confirmed by the results in this study. This premature ageing trait was ameliorated to an extent with some of the NADES extracts (CiAPD14 propolis) and further provided us with confidence to study the underlying mechanisms in detail. These antiageing effects varied in the different combinations studied and in the different mutant cells, for some of which like the single *hho1delta* mutant we did not observe a correlation between the CLS survival on the last time point and the expression of *CDC28*. In contrast, supplementation of these cells with the pure solvent at the 72^nd^-hour time point led to a correlation with *CDC28* expression levels, thus suggesting that the phenol compounds in the CiAPD14 extracts, especially propolis, exerted oxidative stress [69]. In general, the NADES CiAPD14 propolis and CiAPD14 *S. scardica* extracts had a stronger antiageing effect compared to the CiAPD14 *P. major* extract. We associated these results with the phenolic content (Table 2) where the CiAPD14 propolis extract had the highest content, followed by the CiAPD14 *S. scardica* and CiAPD14 *P. major* ones. We, therefore, assume that future developments and applications of NADES plant extracts in antiageing interventions need extensive research in the light of their phenolic content and their biological underlying mechanisms, some of which even induction oxidative stress.

Our future perspectives lie in the anticipation of a more detailed study of the effects of these NADES solvents and extracts on higher eukaryotic model systems. This will shed light on the molecular mechanisms triggered by these NADES solvents and extracts and will allow systematic studies among species on their antiageing features.

## Figures and Tables

**Table 1:** RT-qPCR primer pairs.

Oligomer name	Sequence 5′–3′	Amplicon length (bp)
Sc*ACT1*_For	CGGTAACATCGTTATGTCCGGTG	93
Sc*ACT1*_Rev	ATGGAAGATGGAGCCAAAGCG	
Sc*CDC28*_For	AGGAAACCAATCTTCAGTGGCGA	91
Sc*CDC28*_Rev	CTGGCCATATAGCTTCATTCGGC	
Sc*RAD9*_For	GCATGTTTGAGCGCAGGTAG	102
Sc*RAD9*_Rev	TCTGGGTACTAAAGAATCTAAGGCA	

**Table 2:** Concentrations at which the studied NADES solvent CiAPD14 and the respective propolis and plant extracts were used for culture media supplementation of the yeast cells during their CLS. The concentrations were chosen, based on the data collected in our previously published research [19].

Solvent/extract	Final concentrations (f.c.) (%*v*/*v*, *μ*L in 100 *μ*L)	Total phenolic content in the used concentrations (*μ*g/mL)
CiAPD14	0.089	—
CiAPD14 propolis	0.027	3.3048
CiAPD14 *S. scardica*	0.046	0.8878
CiAPD14 *P. major*	0.045	0.459

## Data Availability

All data supporting this study are freely available.

## References

[1] Shon M. S., Lee Y., Song J. H. (2014). Anti-aging potential of extracts prepared from fruits and medicinal herbs cultivated in the Gyeongnam area of Korea. *Preventive Nutrition and Food Science*.

[2] Hodgson R., Kennedy B. K., Masliah E. (2020). Aging: therapeutics for a healthy future. *Neuroscience and Biobehavioral Reviews*.

[3] Troen B. R. (2003). The biology of aging. *Mount Sinai Journal of Medicine*.

[4] Liochev S. I. (2015). Which is the most significant cause of aging?. *Antioxidants*.

[5] Jin K. (2010). Modern biological theories of aging. *Aging and Disease*.

[6] Sahabi S., Jafari-Gharabaghlou D., Zarghami N. (2022). A new insight into cell biological and biochemical changes through aging. *Acta Histochemica*.

[7] Vujin A., Dick K. (2020). The information theory of aging. *Health Science Inquiry*.

[8] Hayano M., Yang J.-H., Bonkowski M. (2019). DNA break-induced epigenetic drift as a cause of mammalian aging. *SSRN Electronic Journal*.

[9] Sinclair D. A., LaPlante M. D., Delphia C. L. (2019). *Lifespan: Why We Age - and Why We Don’t Have to*.

[10] Chopra A. S., Lordan R., Horbańczuk O. K. (2022). The current use and evolving landscape of nutraceuticals. *Pharmacological Research*.

[11] Paiva A., Craveiro R., Aroso I., Martins M., Reis R. L., Duarte A. R. C. (2014). Natural deep eutectic solvents–solvents for the 21st century. *ACS Sustainable Chemistry & Engineering*.

[12] Ivanović M., Islamčević Razboršek M., Kolar M. (2020). Innovative extraction techniques using deep eutectic solvents and analytical methods for the isolation and characterization of natural bioactive compounds from plant material. *Plants*.

[13] Liu Y., Friesen J. B., McAlpine J. B., Lankin D. C., Chen S. N., Pauli G. F. (2018). Natural deep eutectic solvents: properties, applications, and perspectives. *Journal of Natural Products*.

[14] Choi Y. H., van Spronsen J., Dai Y. (2011). Are natural deep eutectic solvents the missing link in understanding cellular metabolism and physiology?. *Plant Physiology*.

[15] Oomen W. W., Begines P., Mustafa N. R., Wilson E. G., Verpoorte R., Choi Y. H. (2020). Natural deep eutectic solvent extraction of flavonoids of Scutellaria baicalensis as a replacement for conventional organic solvents. *Molecules*.

[16] Ruesgas-Ramon M., Figueroa-Espinoza M. C., Durand E. (2017). Application of deep eutectic solvents (DES) for phenolic compounds extraction: overview, challenges, and opportunities. *Journal of Agricultural and Food Chemistry*.

[17] Ünlü A. E. (2021). Green and non-conventional extraction of bioactive compounds from olive leaves: screening of novel natural deep eutectic solvents and investigation of process parameters. *Waste and Biomass Valorization*.

[18] da Silva D. T., Rodrigues R. F., Machado N. M. (2020). Natural deep eutectic solvent (NADES)-based blueberry extracts protect against ethanol-induced gastric ulcer in rats. *Food Research International*.

[19] Grozdanova T., Trusheva B., Alipieva K. (2020). Extracts of medicinal plants with natural deep eutectic solvents: enhanced antimicrobial activity and low genotoxicity. *BMC Chemistry*.

[20] Torres-Vega J., Gómez-Alonso S., Pérez-Navarro J., Alarcón-Enos J., Pastene-Navarrete E. (2021). Polyphenolic compounds extracted and purified from Buddleja globosa Hope (Buddlejaceae) leaves using natural deep eutectic solvents and centrifugal partition chromatography. *Molecules*.

[21] Rukavina I., Rodrigues M. J., Pereira C. G. (2021). Greener is better: first approach for the use of natural deep eutectic solvents (NADES) to extract antioxidants from the medicinal halophyte Polygonum maritimum L. *Molecules*.

[22] Bhattacharya T., Dey P. S., Akter R., Kabir M. T., Rahman M. H., Rauf A. (2021). Effect of natural leaf extracts as phytomedicine in curing geriatrics. *Experimental Gerontology*.

[23] Kratchanova M., Denev P., Ciz M., Lojek A., Mihailov A. (2010). Evaluation of antioxidant activity of medicinal plants containing polyphenol compounds. Comparison of two extraction systems. *Acta Biochimica Polonica*.

[24] Rahman M. M., Rahaman M. S., Islam M. R. (2022). Role of phenolic compounds in human disease: current knowledge and future prospects. *Molecules*.

[25] Dos Santos Á., Toseland C. P. (2021). Regulation of nuclear mechanics and the impact on DNA damage. *International Journal of Molecular Sciences*.

[26] Roger L., Tomas F., Gire V. (2021). Mechanisms and regulation of cellular senescence. *International Journal of Molecular Sciences*.

[27] Pathak R. U., Soujanya M., Mishra R. K. (2021). Deterioration of nuclear morphology and architecture: a hallmark of senescence and aging. *Ageing Research Reviews*.

[28] Pegoraro G., Misteli T. (2009). The central role of chromatin maintenance in aging. *Aging (Albany NY)*.

[29] Liu B., Yip R., Zhou Z. (2012). Chromatin remodeling, DNA damage repair and aging. *Current Genomics*.

[30] Miloshev G., Staneva D., Uzunova K. (2019). Linker histones and chromatin remodelling complexes maintain genome stability and control cellular ageing. *Mechanisms of Ageing and Development*.

[31] Vasileva B., Staneva D., Krasteva N., Miloshev G., Georgieva M. (2021). Changes in chromatin organization eradicate cellular stress resilience to UVA/B light and induce premature aging. *Cell*.

[32] Fabrizio P., Longo V. D. (2003). The chronological life span of Saccharomyces cerevisiae. *Aging Cell*.

[33] Longo V. D., Shadel G. S., Kaeberlein M., Kennedy B. (2012). Replicative and chronological aging in _Saccharomyces cerevisiae_. *Cell Metabolism*.

[34] Fontana L., Partridge L., Longo V. D. (2010). Extending healthy life span--from yeast to humans. *Science*.

[35] Fatouros C., Pir G. J., Biernat J. (2012). Inhibition of tau aggregation in a novel Caenorhabditis elegans model of tauopathy mitigates proteotoxicity. *Human Molecular Genetics*.

[36] Huang Z., Chen K., Zhang J. (2013). A functional variomics tool for discovering drug-resistance genes and drug targets. *Cell Reports*.

[37] Zhang X., Smith D. L., Meriin A. B. (2005). A potent small molecule inhibits polyglutamine aggregation in Huntington’s disease neurons and suppresses neurodegenerationin vivo. *Proceedings of the National Academy of Sciences of the United States of America*.

[38] Kaeberlein M. (2010). Lessons on longevity from budding yeast. *Nature*.

[39] Sherman F. (2002). Getting started with yeast. *Methods in Enzymology*.

[40] Tenreiro S., Munder M. C., Alberti S., Outeiro T. F. (2013). Harnessing the power of yeast to unravel the molecular basis of neurodegeneration. *Journal of Neurochemistry*.

[41] Boone C., Bussey H., Andrews B. J. (2007). Exploring genetic interactions and networks with yeast. *Nature Reviews. Genetics*.

[42] Muller I., Zimmermann M., Becker D., Flomer M. (1980). Calendar life span _versus_ budding lifespan of _Saccharomyces cerevisiae_. *Mechanisms of Ageing and Development*.

[43] An J. Y., Kim C., Park N. R. (2022). Clinical anti-aging efficacy of propolis polymeric nanoparticles prepared by a temperature-induced phase transition method. *Journal of Cosmetic Dermatology*.

[44] Anjum S. I., Ullah A., Khan K. A. (2019). Composition and functional properties of propolis (bee glue): a review. *Saudi Journal of Biological Sciences*.

[45] Tadic V. M., Jeremic I., Dobric S. (2012). Anti-inflammatory, gastroprotective, and cytotoxic effects ofSideritis scardicaextracts. *Planta Medica*.

[46] Todorova M., Trendafilova A. (2014). _Sideritis scardica_ Griseb., an endemic species of Balkan peninsula: traditional uses, cultivation, chemical composition, biological activity. *Journal of Ethnopharmacology*.

[47] Farcas A. D., Mot A. C., Parvu A. E. (2019). In vivo pharmacological and anti-inflammatory evaluation of xerophyte Plantago sempervirens Crantz. *Oxidative Medicine and Cellular Longevity*.

[48] Georgieva M., Staneva D., Uzunova K. (2015). The linker histone in _Saccharomyces cerevisiae_ interacts with actin-related protein 4 and both regulate chromatin structure and cellular morphology. *The International Journal of Biochemistry & Cell Biology*.

[49] Harata M., Zhang Y., Stillman D. J. (2002). Correlation between chromatin association and transcriptional regulation for the Act3p/Arp4 nuclear actin-related protein of Saccharomyces cerevisiae. *Nucleic Acids Research*.

[50] Georgieva M., Roguev A., Balashev K., Zlatanova J., Miloshev G. (2012). Hho1p, the linker histone of _Saccharomyces cerevisiae_ , is important for the proper chromatin organization _in vivo_. *Biochimica et Biophysica Acta*.

[51] Georgieva M., Staneva D., Uzunova K., Miloshev G. (2012). The deletion of the gene for the linker histone inARP 4mutant yeast cells is not deleterious. *Biotechnology & Biotechnological Equipment*.

[52] Livak K. J., Schmittgen T. D. (2001). Analysis of relative gene expression data using real-time quantitative PCR and the 2^−*ΔΔ* _C_^_T_ method. *Methods*.

[53] Uzunova K., Georgieva M., Miloshev G. (2013). Saccharomyces cerevisiae linker histone—Hho1p maintains chromatin loop organization during ageing. *Oxidative Medicine and Cellular Longevity*.

[54] Longo V. D., Fabrizio P. (2012). Chronological aging in Saccharomyces cerevisiae. *Sub-Cellular Biochemistry*.

[55] Galdieri L., Mehrotra S., Yu S., Vancura A. (2010). Transcriptional regulation in yeast during diauxic shift and stationary phase. *OMICS*.

[56] Mohammad K., Titorenko V. I. (2018). Yeast chronological aging is linked to cell cycle regulation. *Cell Cycle*.

[57] Zhang H., Siede W. (2004). Analysis of the budding yeast Saccharomyces cerevisiae cell cycle by morphological criteria and flow cytometry. *Methods in Molecular Biology*.

[58] Gire V., Dulic V. (2015). Senescence from G2 arrest, revisited. *Cell Cycle*.

[59] Allen C., Büttner S., Aragon A. D. (2006). Isolation of quiescent and nonquiescent cells from yeast stationary-phase cultures. *The Journal of Cell Biology*.

[60] Li L., Miles S., Melville Z., Prasad A., Bradley G., Breeden L. L. (2013). Key events during the transition from rapid growth to quiescence in budding yeast require posttranscriptional regulators. *Molecular Biology of the Cell*.

[61] Zimmermann C., Chymkowitch P., Eldholm V. (2011). A chemical-genetic screen to unravel the genetic network of &lt; em&gt; CDC28/CDK1&lt;/em&gt; links ubiquitin and Rad6–Bre1 to cell cycle progression. *Proceedings of the National Academy of Sciences*.

[62] Henderson K. A., Kee K., Maleki S., Santini P. A., Keeney S. (2006). Cyclin-dependent kinase directly regulates initiation of meiotic recombination. *Cell*.

[63] Ira G., Pellicioli A., Balijja A. (2004). DNA end resection, homologous recombination and DNA damage checkpoint activation require CDK1. *Nature*.

[64] Aylon Y., Liefshitz B., Kupiec M. (2004). The CDK regulates repair of double-strand breaks by homologous recombination during the cell cycle. *The EMBO Journal*.

[65] Downs J. A., Kosmidou E., Morgan A., Jackson S. P. (2003). Suppression of homologous recombination by the _Saccharomyces cerevisiae_ linker histone. *Molecular Cell*.

[66] Lieberman H. B. (2006). Rad9, an evolutionarily conserved gene with multiple functions for preserving genomic integrity. *Journal of Cellular Biochemistry*.

[67] Ciccia A., Elledge S. J. (2010). The DNA damage response: making it safe to play with knives. *Molecular Cell*.

[68] Tang S. C., Yang J. H. (2018). Dual effects of alpha-hydroxy acids on the skin. *Molecules*.

[69] Tsai Y. C., Wang Y. H., Liou C. C., Lin Y. C., Huang H., Liu Y. C. (2012). Induction of oxidative DNA damage by flavonoids of propolis: its mechanism and implication about antioxidant capacity. *Chemical Research in Toxicology*.

